# Unlocking the potential of cardiac TRP channels using knockout mice models

**DOI:** 10.3389/fphys.2025.1585356

**Published:** 2025-04-17

**Authors:** Kanchan Kulkarni, Richard D. Walton, Sebastien Chaigne

**Affiliations:** ^1^ IHU Liryc, INSERM, U1045, CRCTB, University Bordeaux, Bordeaux, France; ^2^ CHU de Bordeaux, Cardiology, INSERM, U1045, CRCTB, Bordeaux, France

**Keywords:** TRP channels, knockout (KO) mice, cardiac physiology, cardiac pathology, calcium homeostasis

## Introduction

Cardiovascular disease remains the leading global cause of mortality, underscoring the urgent need to better understand the mechanisms driving cardiac dysfunction (Jagannathan et al., 2019). Ion channels are key regulators of cardiac excitability, and among them, the transient receptor potential (TRP) family has gained attention for its diverse physiological roles. TRP channels are ubiquitously expressed in mammalian hearts and function as essential “cellular switches” that respond to a wide range of chemical and physical stimuli, influencing sensory physiology (Hof et al., 2019; Zhang et al., 2023). Comprising 28 members across six subfamilies, TRP channels act as polymodal sensors responding to chemical, thermal, and mechanical cues. In the heart, these channels influence calcium (Ca^2+^) dynamics, electrical conduction, mechanical responsiveness, and stress adaptation (Hof et al., 2019).

Building on the foundational discoveries by Drs. David Julius and Ardem Patapoutian on temperature and mechanotransduction pathways—work that earned the 2021 Nobel Prize in Physiology or Medicine—there is growing recognition of TRP channels’ roles beyond sensory systems, including cardiovascular physiology and pathophysiology (Caterina et al., 1997; Peier et al., 2002). In particular, genetically modified knockout (KO) mice models have emerged as powerful tools to explore TRP channel functions in cardiac tissue, allowing for precise dissection of their mechanistic roles in disease progression and therapeutic potential. This opinion letter highlights key advances and future directions in further leveraging TRP KO models to unlock the therapeutic promise of TRP channels in cardiac health.

## Cardiac TRP channels: multifaceted roles and research frontiers

TRP channels regulate numerous cardiac functions, including rhythm generation, Ca^2+^ handling, contractility, and responses to mechanical stress (Hof et al., 2016; Chaigne et al., 2023; Hu et al., 2023; Veteto et al., 2020). Several family members (e.g., TRPC6, TRPM4, TRPV4) have been implicated in arrhythmogenesis, hypertrophy, fibrosis, and ischemia-reperfusion injury. Their broad permeability to cations and ability to integrate diverse stimuli render them critical for both normal physiology and pathological remodeling (Numata et al., 2016; Guo et al., 2024). In physiological conditions, inward current through these channels is primarily due to the entry of Ca^2+^, sodium (Na^+^), or magnesium (Mg^2+^), while outward current results from potassium (K^+^) exiting the cell. Notably, very little is known about Mg^2+^ handling in relation to TRP channel activities and heart function, even though mutations in sub-families of these channels have been shown to be associated with disturbances in Mg^2+^, highlighting the need for further investigations (Gwanyanya et al., 2004; Jin et al., 2022).

Activation of TRP channels typically induces a depolarizing current because the main flux of cations is inward. This characteristic makes TRP channels particularly relevant in excitable cells like cardiomyocytes, where they can prolong action potential duration, as observed following activation or potentiation of TRPC3 (Ju et al., 2015), TRPM4 (permeable to Na^+^) (Hof et al., 2016; Simard et al., 2013), TRPM6-7 (Gwanyanya et al., 2021) and TRPV4 (Chaigne et al., 2023). However, many TRP channels do not function as classical mechanosensors. Recent studies show that while TRPs may not be directly gated by membrane stretch (O'Neil and Heller, 2005; Nikolaev et al., 2019), they can act as mechano-effectors downstream of primary mechanosensors, including PIEZO1 channels (Guo et al., 2024; Guo et al., 2021; Yu et al., 2022). For instance, TRPV4 interacts with volume-sensitive signalling molecules such as Src kinase (Zou et al., 2022) and phospholipase A2 (Gorelick and Nathanson, 2020). These emerging insights necessitate a more nuanced classification of TRP channels in mechanotransduction.

Importantly, the effector role of TRP channels—particularly their position downstream of primary sensors—may open opportunities for more progressive and targeted therapeutic interventions. Rather than blocking upstream mechanotransduction entirely, targeting TRPs may allow fine-tuning of maladaptive downstream signalling. This approach could offer graded, context-sensitive modulation in heart failure and hypertrophy, with lower risk of disrupting physiological homeostasis. Supporting this concept, TRPC3 and TRPC6, for example, have been shown to act as calcium-permeable effectors that activate the calcineurin–NFAT pathway downstream of neurohumoral and mechanical stimuli (Seo et al., 2014). Inhibiting such TRPs may thus interrupt pro-hypertrophic signalling while preserving upstream sensory mechanisms essential for baseline cardiac function. This positions TRP channels as viable and adaptable targets for therapeutic strategies focused on disease modification without systemic disruption.

However, selective agonists and antagonists for TRP channels are scarce, with only a few known agents such as capsaicin, an agonist of TRPV1 (Caterina et al., 1997; Wang and Wang, 2005), that have undergone comprehensive pharmacological evaluation. Hence, while this approach could offer graded, context-sensitive modulation in heart failure and hypertrophy, with lower risk of disrupting physiological homeostasis, further studies are required to identify selective modulators of these channels. The use of TRP KO mice offers a potent avenue to better understand the specific roles of this ion channel family and validate the effectiveness and safety of their targeted modulators.

## Advantages of knockout models in TRP research

Given the limited availability and selectivity of pharmacological modulators for TRP channels, KO mice models provide an essential approach for understanding cardiac channel-specific functions. For example, TRPM4 KO mice have clarified this channel’s role in prolonging action potentials and promoting arrhythmia in stress models (Guinamard et al., 2015). Similarly, both TRPC6 and TRPV4 KO mice resist pressure-overload-induced hypertrophy (Jia et al., 2024; Kinoshita et al., 2010; Tang et al., 2022). Interestingly, TRPM4 has emerged as a critical integrator of distinct pathological stimuli. Studies have shown that both pressure overload and Angiotensin II (AngII) stimulation activate TRPM4 through different signalling pathways (Guo et al., 2021; Kecskes et al., 2015). This convergence highlights TRPM4’s central role in cardiac disease and underscores its promise as a therapeutic target across multiple disease contexts.

A comprehensive summary of current TRP KO mice models is presented in Table 1, demonstrating the scope and complexity of the roles of these channels in cardiovascular pathophysiology. KO models offer several advantages:

• Causality: Genetic deletion isolates the contribution of individual channels to disease phenotypes.
• Compensation Insight: TRP families exhibit overlapping roles. KO studies have revealed compensatory expression among TRPC channels (Vandewauw et al., 2018), highlighting complex redundancy.
• Therapeutic Discovery: By testing drugs in KO *versus* wild-type mice, researchers can assess target specificity and identify off-target effects.

**TABLE 1:** TRP channel expression and properties in myocytes.

TRP channels	Myocytes expression	Myocytes current	KO TRP model	Inhibitor	Cardiac function	Mechanosensitive channels	Cardiac pathological implication	Mutation
TRPA1 (Conklin et al., 2019; Paulsen et al., 2015)	Ventricles*	left ventricle*	Yes (rat)	HC-030031	Ventricular inotropy and lusitropy	SAC*	Myocardial I/R, arrhythmia	Unknown
TRPC1 (Shenton and Pyner, 2014; Liao et al., 2013)	NS, atrium, ventricles, PFs	Yes atrial and ventricular	Yes (mouse)	D-GsMTx4	Cardiac hypertrophy and osmolarity	SAC	Cardiac hypertrophy and arrhythmia (Seth et al., 2009; Ohba et al., 2017)	Unknown
TRPC2	mRNA in SN & ventricles	Not extracted from myocytes	Yes (mouse)	—	—	—	Unknown	Unknown
TRPC3	Atrium and ventricles	TRPC Like current neonatal-myocytes, rat	Yes (mouse)	SAR7334	Cardiac hypertrophy	SAC (link to TRPC6)	Ang II-induced cardiac hypertrophy, excessive ROS production, AF & fibrosis (Kitajima et al., 2016; Doleschal et al., 2015; Han et al., 2016; Brenner and Dolmetsch, 2007; Harada et al., 2012)	Unknown
TRPC4 (Duan et al., 2018a; Vinayagam et al., 2020)	SN, Atrium and ventricles	Not extracted from myocytes	Yes (mouse)	HC-069	Ca^2+^ homeostasis	Not mechanosensitive ion channel	Cardiac hypertrophy (no link with the Ang II pathway) (Jung and Kittleson, 2011; Camacho Londono et al., 2015)	(missense SNP - gain of function)
TRPC5 (Yin et al., 2024; Wright et al., 2020)	Atrium & Ventricles	Not extracted from myocytes	Yes (mouse)	HC-070	Cardiac hypertrophy	VAC & SAC	Cardiac hypertrophy, apoptosis, oxidative stress, I/R injury, hypertension, heart failure, arrhythmia	Unknown
TRPC6 (Urban et al., 2016; Hafner et al., 2019)	SN, atrium and ventricles	TRPC Like current neonatal-myocytes, rat	Yes (mouse)	Larixyl acetate -SAR7334	Cardiac hypertrophy/fibrosis, Ca^2+^ homeostasis and arrhythmogenesis	SAC	Ang II-induced cardiac hypertrophy (Onohara et al., 2006; Bush et al., 2006)	Unknown
TRPC7 (Maier et al., 2015; Liu et al., 2021)	Atrium	Yes (neonatal-myocytes, rat)	Yes (mouse)	SAR7334	Apoptosis	Not mechanosensitive ion channel	Ang II/endotholin1-induced cardiac hypertrophy and apoptosis (Satoh et al., 2007)	Unknown
TRPM1 (Lambert et al., 2011)	—	—	Yes (mouse)	—	—	—	Unknown	Unknown
TRPM2 (Takahashi et al., 2012; Yin et al., 2019; Miller et al., 2014; Luo et al., 2018)	Ventricles	Yes (ventricular)	Yes (mouse)	3-MFA	Cardiac metabolism	Not classified as a mechano-gated ion channel	Mitochondrial dysfunction, cardiac ischemic injury and fibrosis during AF (Chen et al., 2013; Miller et al., 2013)	Unknown
TRPM3 (Zhao and MacKinnon, 2023)	mRNA presence in atria and ventricles	—	Yes (mouse)	Isosakuranetin or primidone	Not defined within cardiomyocytes	Not classified as a mechano-gated ion channel	Cardiac hypertrophy and Mitochondrial Ca^2+^ overload	Unknown
TRPM4 (Guinamard et al., 2006; Feng et al., 2021; Guo et al., 2017)	SN, Atrium, Ventricles & Purkinje	Yes (SN, atrial, Purkinje and ventricular	Yes (mouse)	9-Phenanthrol, Flufenamic acid	Cardiac action potential and conduction	Not classified as a mechano-gated ion channel	Arrhythmias, hypoxia–reoxygenation injuries and negative or positive regulators for angiotensin II and pressure overload induced cardiac hypertrophy (Guo et al., 2021; Kecskes et al., 2015; Hof et al., 2017; Du et al., 2010; Burt et al., 2013; Chen et al., 2022)	Brs, conduction block and long QT syndrome
TRPM5	—	—	Yes (mouse)	Triphenylpho-sine oxide (TPPO), Flufenamic acid	—	Not classified as a mechano-gated ion channel		
TRPM6	Atrium	Not extracted from myocytes	Yes (mouse - heterozygous)	Mesendogen	Cardiac automaticity	Not classified as a mechano-gated ion channel	Sinus tachycardia, arrhythmia and fibrosis (Zhang et al., 2015)	Unknown
TRPM7 (Jin et al., 2008; Li et al., 2007; Sah et al., 2013; Duan et al., 2018b)	SN, Atrium & Ventricles	Yes (atrial and ventricular)	Yes (mouse)	Waixinecin A	Fibroblast proliferation, differentiation, Heart development, automaticity and conduction	VAC (hypertonic stress) & SAC	Fibrosis (Du et al., 2010; Li et al., 2017; Yu et al., 2014)	Long QT syndrome
TRPM8 (Cheng et al., 2019; Yin et al., 2024)	Rat myocardium	Not extracted from myocytes	Yes (mouse)	Large panel	Cardiac apoptosis, cardioprotector and I/R injury	No classified as a mechano-gated ion channel	Myocardial I/R (Cheng et al., 2019)	Unknown
TRPV1 (Wang and Wang, 2005; Liao et al., 2013)	Only mRNA in ventricles	Not extracted from myocytes	Yes (mouse)	Capzazepine	—	Unknown in the heart	Cardiac hypertrophy and cardiomyopathies	Unknown
TRPV2 (Zubcevic et al., 2016)	Ventricles	Not extracted from myocytes	Yes (mouse)	Tranilast (or SKF96365)	Structural andfunctional protein	VAC	Contractility, myocardial damages, fibrosis (Katanosaka et al., 2014)	Unknown
TRPV3 (Zhang et al., 2018; Singh et al., 2018; Shimada et al., 2020)	Ventricles	Not extracted from myocytes	Yes (mouse)	Ruthenium red (nonselective)	Cardiac hypertrophy	Unknown in the heart	Ang II-induced cardiac hypertrophy and fibrosis (Zhang et al., 2018)	Unknown
TRPV4 (Jones et al., 2019; Liedtke et al., 2003; Suzuki et al., 2003)	Atrium and ventricles	Not extracted from myocytes	Yes (mouse)	GSK219 HC-067047	Electrical activity & Ca^2+^ homeostasis	VAC & SAC	Fibrotic and functional responses of the heart to pressure overload, arrhythmogenesis, cardiac hypertrophy and myocardial damages (Veteto et al., 2020; Jones et al., 2019; Peana et al., 2022)	Unknown
TRPV5 (Fluck et al., 2022; Hughes et al., 2019)	—	—	Yes (mouse)	ZINC 17988990	—	Not classified as a mechano-gated ion channel	Unknown	Unknown
TRPV6 (Neuberger et al., 2021; Saotome et al., 2016; Chen et al., 2014)	—	—	Yes (mouse)	ZINC 17988991	—	Not classified as a mechano-gated ion channel	Unknown	Unknown
TRPP1	Ventricles	Not extracted from myocytes	No	unspecific TRPP1/V2: amiloride	Ca^2+^ homeostasis, development and cardioprotection	—	Cardiac hypertrophy and malformation (Fick et al., 1995)	HF
TRPP2 (Li et al., 2015)	Ventricles	Yes (ventricular)	Yes (mouse)	MK-870 hydrochloride	Cardiac hypertrophy, valve development and inflammation	—	Cardiac malformation, cardiomyopathy and valvular dysfunction (Fick et al., 1995)	HF
TRPP3 (Lu et al., 2018)	Ventricles	Not extracted from myocytes	Yes (mouse)	Phenamil methanesulfonate	Ca^2+^ homeostasis and cardiac hypertrophy	—	Cardiac hypertrophy and heart failure	Unknown

*Disputed; SN, sinus node; VAC, volume activated channels; SAC, stretch activated channels; HF, heart failure; I/R, ischemia/reperfusion injury; Note that TRPM4 and TRPM5 are permeable to sodium and potassium (Launay et al., 2002); Brs, Brugada syndrome; ROS, reactive oxygen species; Ang II, Angiotensin II, AF, atrial fibrillation.

These benefits make TRP KO models a cornerstone of mechanistic cardiovascular research.

KO mice models offer an invaluable tool for uncovering new components of cellular dysfunction and their translational impacts. While clinical electrocardiogram (ECG) features such as heart rate, PR interval, QRS complex, and QT/QTc interval, are crucial for diagnosing and monitoring physiological and various cardiac conditions, *in-vitro* parameters, offer focused, detailed insights into the cellular and molecular mechanisms underlying cardiac electrical activity. Unitary currents, measured through single-channel recordings, reveal the behaviour of individual ion channels that contribute to the macroscopic currents linked to cellular activation profiles (Guinamard et al., 2015). For example, TRP channels dysfunction detected through unitary current recordings, can be correlated with changes in both action potential and ECG parameters (Chaigne et al., 2021), indicating a risk of arrhythmias.

While it has been suggested that mammalian TRP channels are insensitive to membrane stretch, some TRP channels respond to mechanical forces (Liu and Montell, 2015) applied to the cell membrane from external influences (see Table 1). These forces can modulate the open probability of the channels, without involving a signalling cascade. This mechanosensitivity allows TRP channels to respond to various physical stimuli such as pressure, stretch, and shear stress, thereby playing a crucial role in various physiological processes (Liu and Montell, 2015; Moran et al., 2004). KO models have been effective in investigating the mechanosensitivity of TRP channels and their down-stream effects. Furthermore, using KO mice, researchers can also explore potential compensatory mechanisms that may arise due to the absence or inhibition of targeted TRP channels. This approach provides insights into the complex interplay between different ion channels and signalling pathways in maintaining physiological homeostasis.

Finally, integrative *in-vivo* models can help uncover systemic effects and potential off-target effects that might not be evident in isolated cardiomyocytes which lack the full spectrum of hormonal (Liu et al., 2022), immune (Wu et al., 2023), and nervous system regulation (Shanks et al., 2019). For instance, a drug might show promise *in-vitro* but could interact with other physiological systems *in-vivo*, leading to unforeseen side-effects. Comprehensive *in-vivo* testing can thus, identify early issues in the drug development process, saving time and resources. Through transesophageal stimulation, which enables the analysis of underlying mechanisms of cardiac rhythm disorders across the heart’s chambers, the use of KO models, helps to indirectly establish a link between the absence of a gene and its involvement in the development of arrhythmia. Note that this approach may not be ideal for mechanical ion channels that are sensitive to stretching. Ultimately, the absence of the gene of interest can indirectly enhance our understanding of the mechanisms underlying cardiac tissue hypertrophy and fibrosis (Guo et al., 2021; Zou et al., 2022; Watanabe et al., 2013).

## Future directions and strategic areas for therapeutic insight

Several innovative approaches are currently under development and have the potential to pioneer change in cardiac research. A key strategy involves structure-based drug design, which aims to create highly specific inhibitors by targeting the three-dimensional TRP channels structure. Another promising technique, Surface Plasmon Resonance (SPR), provides real-time insights into how these inhibitors interact with TRP channels, enhancing our understanding of their binding mechanisms. Their thorough investigation could lead to new therapeutic approaches for treating cardiovascular abnormalities. Main takeaways that could influence future therapeutic strategies are outlined below:

1. Mechanotransduction and Heart Failure: Mechanosensitive TRP channels (e.g., TRPC6 (Onohara et al., 2006), TRPM7 (Yu et al., 2014), TRPV4 (Veteto et al., 2020)) are activated under pathological stretch, making them promising targets in heart failure. KO models subjected to pressure overload can dissect these channels’ contributions to maladaptive remodelling.
2. Sudden Cardiac Death and Arrhythmia: TRPA1 (Conklin et al., 2019) and TRPM4 (Vandewiele et al., 2022; Yang et al., 2006) KO mice exhibit resistance to arrhythmia under stress or ischemic conditions. TRP channels modulating action potential duration are ideal candidates for anti-arrhythmic drug development.
3. Inter-individual Variability in Drug Response: KO models help reveal how TRP channel expression variability influences therapeutic outcomes. For example, TRPC3 (Kitajima et al., 2016) and TRPM2 are stress-responsive and may contribute to variable responses to oxidative or hypertrophic stimuli (Onohara et al., 2006; Kitajima et al., 2016; Takahashi et al., 2012; Chen et al., 2013).
4. Brain-Heart Axis and Neurogenic Modulation: With expression in sensory neurons and cardiac tissue, channels like TRPM8 and TRPV1 (Yoshie et al., 2020) may mediate autonomic effects on the heart (Shanks et al., 2019; Yin et al., 2024). TRP KO models enable investigation into neuro-cardiac interactions and their therapeutic modulation.
5. Translation to Human Therapies: Though interspecies differences exist, KO mice remain critical in screening for TRP-targeted therapies, particularly where pharmacological tools are lacking or non-selective. Caution is needed when extrapolating findings, but the insights remain invaluable.

## Limitations and integration with complementary models

While KO mice are indispensable, they have limitations. Genetic deletion can trigger compensatory mechanisms, masking functional deficits. Moreover, mouse cardiac electrophysiology differs from humans in ion channel expression and repolarization dynamics (Joukar, 2021). These differences, including variations in immune system responses (Gilbertson and Weinmann, 2021), can undeniably impact the relevance of findings to human clinical settings. In this case, human tissue or cellular models, offer promising alternatives. Additionally, while not all TRP channels can compensate for one another, functional redundancy within the TRP family has been documented. For example, research has shown that the presence of at least one of these channels (TRPA1, TRPM3 or TRPV1) helps preserve somatosensory heat responsiveness (Vandewauw et al., 2018). These compensatory effects must be carefully considered when interpreting KO model data.

To address these challenges, future studies could incorporate the following complementary strategies:

• Integrate human iPSC-derived cardiomyocytes and organoids
• Employ computational modelling of TRP-mediated currents
• Combine TRP KOs with spatial omics and functional imaging

These approaches could provide a comprehensive understanding of TRP channel function across species and contexts.

## Conclusion

Cardiac TRP channels are emerging as pivotal regulators of cardiovascular physiology and pathology. KO mice models offer a unique opportunity to define their functions, explore disease mechanisms, and identify new therapeutic strategies. By refining our understanding of TRP channel biology, particularly through the lens of mechanotransduction and electrophysiology, the next-generation of targeted interventions may be realized. We advocate for a continued, strategic use of TRP KO models as a springboard for precision cardiovascular medicine.
